# *Sanctuary*: a *Starship* transposon facilitating the movement of the virulence factor ToxA in fungal wheat pathogens

**DOI:** 10.1128/mbio.01371-25

**Published:** 2025-09-04

**Authors:** Angus Bucknell, Hannah M. Wilson, Karen C. Gonçalves dos Santos, Steven Simpfendorfer, Andrew Milgate, Hugo Germain, Peter S. Solomon, Adam Bentham, Megan C. McDonald

**Affiliations:** 1 School of Biosciences, Institute of Microbiology and Infection, University of Birmingham 1724, Birmingham, United Kingdom; 2 The Sainsbury Laboratory, University of East Anglia, Norwich Research Park, Norfolk, United Kingdom; 3 Division of Plant Sciences, Research School of Biology, The Australian National University, Canberra, Australia; 4 Department of Chemistry, Biochemistry, Physics and Forensic Sciences, Université du Québec à Trois-Rivières 14847, Trois-Rivières, Québec, Canada; 5 NSW Department of Primary Industries and Regional Development, Tamworth Agricultural Institute 56358, Tamworth, Australia; 6 NSW Department of Primary Industries and Regional Development, Wagga Wagga Agricultural Institute 95736, Wagga Wagga, Australia; Cornell University, Ithaca, New York, USA

**Keywords:** *Starships*, ToxA, horizontal gene transfer, *Bipolaris*, transposons, long-read sequencing

## Abstract

There is increasing evidence that mobile genetic elements can drive the emergence of pathogenic fungal species by moving virulence genes horizontally. The 14 kbp *ToxhAT* transposon was shown to move the necrotrophic effector, *ToxA,* horizontally between wheat pathogens, namely *Parastagonospora nodorum*, *Pyrenophora tritici-repentis*, and *Bipolaris sorokiniana*. All three species utilize the ToxA protein to infect wheat. Previous work found *ToxhAT* in distinct chromosomal positions in two *B. sorokiniana* isolates, indicating that the transposon remains active in this species. Here, we confirm the movement of *ToxhAT* using long-read sequencing of eight new and one previously published *B. sorokiniana* isolates. One event of independent transposition of *ToxhAT* was observed, and target site duplications of “TA” were identified, confirming that this is an active transposon in this species that likely falls into the *Tc1/Mariner* transposon family. We propose renaming this non-autonomous transposon to *ToxTA*. Whole genome analysis revealed that *ToxTA* is a passenger embedded in a much larger, conserved 170–196 kbp mobile genetic element. This element, termed *Sanctuary*, belongs to the newly described *Starship* transposon superfamily. This classification is based on the presence of direct repeats, empty insertion sites, a putative tyrosine recombinase gene, and other features of *Starship* transposons. We also show that *ToxTA* has been independently acquired by two different *Starships*, *Sanctuary* and *Horizon,* which share little to no sequence identity, outside of *ToxTA*. This classification makes *Horizon* and *Sanctuary* part of a growing number of *Starships* involved in the horizontal gene transfer of adaptive genetic material between fungal species.

**IMPORTANCE:**

The work presented here expands our understanding of a novel group of mobile genetic elements called *Starships* that facilitate the horizontal exchange of numerous genes between fungal pathogens. Our analysis shows that *Sanctuary* and *ToxTA* are both active transposons within the *Bipolaris sorokiniana* genome. We also show that the smaller *ToxTA* transposon has been independently acquired by two different *Starships*, namely *Sanctuary* in *B. sorokiniana* and *Horizon* in *Pyrenophora tritici-repentis* and *Parastagonospora nodorum*. Outside of *ToxTA,* these two *Starships* share no sequence identity. The acquisition of *ToxTA* by two different mobile elements in three different fungal wheat pathogens demonstrates how horizontal transposon transfer is driving the evolution of virulence in these important wheat pathogens.

## INTRODUCTION

Horizontal gene transfer (HGT) is the non-Mendelian exchange of genetic material between organisms (1, 2). HGT is a significant driver of rapid adaptation to a changing environment and is of particular concern in microbial pathogens (3). Gene exchange through HGT can rapidly enhance virulence (4), increase host range (5), or increase microbial competitive ability (6). Eukaryotic HGT was once thought to be a rare event due to the complexity of these cell types (7). However, as more genome assemblies became available, especially ones assembled with long-read sequencing technologies, many examples of HGT between eukaryotes have been described (1). Long-read assemblies have also enabled a much more detailed look at the repetitive content of genomes, namely transposons and their history of HGT. While such discoveries were made before the advent of next-generation sequencing, dating back to the 1990s, robust evidence in the form of chromosome-level genome assemblies has uncovered many previously overlooked HGT events (8). These discoveries suggest that transposon-mediated HGT between eukaryotes is an ongoing and evolutionarily ancient form of adaptive evolution, where the underlying mechanism of how these transposons move between organisms remains to be characterized (1, 2).

As opposed to movement between different species, the processes governing transposon movement within a genome are better characterized. Transposons are broadly categorized into two major classes, based on whether they use an RNA (Class I) or DNA (Class II) intermediate to move locations within a genome (9). Other characteristics, such as encoded proteins, structural motifs, and target site length, are used to further divide these transposons into orders, superfamilies, and families. Class I retrotransposons are characterized by transposition involving an RNA intermediate that is reverse transcribed before re-integration into the genome. Class II DNA transposons have no RNA intermediate and move through excision and integration (9). Class II transposition is facilitated by a transposase or recombinase and is often bound by terminal inverted repeats (TIRs) (9, 10). Insertion of a transposon into a new genomic location usually involves a double-stranded break of the DNA that creates short overhangs. The backfilling of these overhangs leads to a duplication of this sequence on either side of the new insertion site, referred to as a target site duplication (TSD). TSDs are another important non-coding feature of both Class I and II transposons. In many instances, these TSDs can be quite difficult to recognize as they range in size from 2 to 8 base pairs. One useful technique to help identify TSDs is to compare the identity of the DNA sequence that directly flanks the insertion to another individual that does not carry the transposon at the same site, often referred to as “empty sites” (10, 11). In any genome assembly, the location of a transposon is only a snapshot of its location in the genome at the time of DNA extraction; however, in the absence of an experimental system where transposon movement can be tracked, annotating TSDs remains an informative method to identify active transposons within a genome.

One well-studied example of eukaryotic HGT is the *ToxA* virulence gene. *ToxA* is found in the fungal wheat pathogens *Pyrenophora tritici-repentis* (12), *Parastagonospora nodorum* (13), and *Bipolaris sorokiniana* (14), all of which cause severe crop losses in wheat crops globally (15, 16). These pathogens can all infect wheat without *ToxA*; however, the presence of this effector has been correlated with more severe disease symptoms, indicating this gene can confer a strong fitness advantage (17). Long-read sequencing was used to demonstrate that *ToxA* is carried within a conserved transposon that was horizontally transferred between *P. nodorum* and *P. tritici-repentis*, and *B. sorokiniana* (18). This transposon, now called *ToxhAT*, is a single-copy, 14 kbp Class II transposon that is found in all three fungal species. *ToxhAT* remains highly conserved with a sequence similarity of ~92% at the nucleotide level, indicative of a very recent HGT event (14). Homologous DNA upstream (61.2 kbp) and downstream (1.7 kbp) of *ToxhAT* is shared between *P. tritici-repentis* and *P. nodorum*, suggesting the HGT event was greater than just *ToxhAT*. However, only *ToxhAT* is shared between *B. sorokiniana* and the other two species (18). Questions such as the order of acquisition by each species and the origins of *ToxhAT* and *ToxA* remain (19).

*ToxhAT* in *P. tritici-repentis* and *P. nodorum* is believed to be inactive due to a high level of repeat-induced point (RIP) mutation that disrupts the TIRs of *ToxhAT* in these species (18). RIP is a mechanism of fungal genome defense where cytosine to thymine (C to T) and guanine to adenine (G to A) polymorphisms are induced (20). Minimal RIP mutation of *ToxhAT* in *B. sorokiniana* has left the transposon largely intact, and recent data from one genome assembly has indicated that *ToxhAT* remains active as it was putatively identified in different chromosomes in different sequenced isolates (18). In *B. sorokiniana* isolate WAI2411, the 14 kbp region of *ToxhAT*, bounded by the TIRs, was inverted and located on a different chromosome in reference to the other two isolates, indicative of active transposition. In another isolate, a roughly 200 kbp region of DNA, including *ToxhAT,* was found translocated to another chromosome, indicative of chromosomal translocation or the movement of a larger mobile genetic element in which *ToxhAT* was embedded. TSDs bounding *ToxhAT* were not identified in any of the three *B. sorokiniana* isolates, which left the question of whether *ToxhAT* remains an active transposon unanswered (18).

While each of the three species that currently harbor *ToxA* has additional mechanisms of pathogenicity, the acquisition of *ToxA* is hypothesized to have enabled the emergence of each species as a significant pathogen of wheat (13). As such, understanding the mechanisms by which *ToxA* is horizontally transferred and/or remains in an active transposon is of great importance for future management of these diseases. In this work, we sequenced and assembled eight new *B. sorokiniana* isolates to explore the hypothesis that *ToxhAT* is an active class II transposon in *B. sorokiniana*. We also drew further comparisons of the genomic regions carrying *ToxhAT* in all three species where it has been identified thus far.

## RESULTS

### Chromosome-level assembly of eight *B. sorokiniana* isolates

Eight novel *ToxA* positive (*ToxA+) B. sorokiniana* isolates were sequenced with the Oxford Nanopore MinION sequencer. The publicly available, fully assembled and annotated genome for the Australian *B. sorokiniana* isolate CS10 (BRIP10943, SAMN05928353) was used as a high-quality reference genome, and the *toxa* negative (*toxa-)* isolate CS27 (BRIP27492, SAMN05928351) was also included for comparative analyses (18). The completeness of each new isolate was scored using the Dothideomycetes Benchmarking Universal Single-Copy Orthologs (BUSCOs), containing 3,786 conserved orthologs (21). QUAST was used to calculate the fraction of the CS10 genome found in each isolate, giving an isolate-specific completeness score (22). The BUSCO and QUAST scores of the novel isolates fell within a narrow range, with a minimum of 93.8% BUSCO score and a minimum QUAST score of 93.5%. A summary of genome assembly completeness for each isolate is outlined in Table 1.

**TABLE 1:** Summary of isolate collection details and genome assembly statistics

Isolate	Collection year	Collection location	Raw data (Gb)	Genome size (Mb)	Complete chromosomes	Total contigs^*b*^	BUSCO score (%)	Quast genome fraction (%)
CS10 (BRIP10943)^*a*^	1966	Biloela, QLD, Australia	N/A^*c*^	36.92		N/A^*c*^	99.6	N/A^*c*^
WAI2411	2015	Nyngan, NSW, Australia	14.20	36.02	10	21	95	94.911
WAI2431	2015	Gilgandra, NSW, Australia	3.13	36.10	8	23	94.5	95.202
WAI2432	2015	North Star, NSW, Australia	10.46	36.47	10	24	94.2	93.252
WAI3285	2017	Inglestone, QLD, Australia	2.99	35.88	6	26	93.8	95.086
WAI3295	2017	Moonie, QLD, Australia	2.84	36.02	4	30	94.5	94.263
WAI3382	2016	Pallamallawa, NSW, Australia	10.09	36.21	11	25	95.7	94.966
WAI3384	2016	Mungindi, QLD, Australia	12.67	36.76	13	21	95.4	93.603
WAI3398	2016	Walgett, NSW, Australia	10.96	36.27	11	23	95.6	93.625

^*a*^ CS10 is an abbreviated name for isolate BRIP10943 (*Cochliobolus sativus* isolate 10).

^*b*^ Total count of all assembled contigs excluding mitochondrial sequences and contigs containing ribosomal repeats only.

^*c*^ N/A, not applicable. Data published previously in reference 18.

Chromosome numbers were assigned to contigs in the new assemblies based on the numbering and orientation of the CS10 reference genome. Whole genome alignments identified chromosomes (Chr) 1–16 and contig (tig) 17 in all new assemblies (Table 1; Table S1). In six of the eight Nanopore isolates, Chr_12 (1.85 Mbp) and tig17 (819 kbp) were present as a single contig totaling 2.5–2.7 Mbp. In CS10, Chr_12 contained two ribosomal repeats at one end, and similarly, tig17 also contained two ribosomal DNA repeats. We confirmed that these two contigs were connected by the rDNA region by aligning the raw reads back to the assembled contigs to verify the Nanopore assemblies manually (Fig. S1A). Using these data, Chr_12 and tig17 were taken to be one chromosome and were concatenated in all final Nanopore assemblies, labeled as Chr_12. Most isolates showed a high degree of synteny to the CS10 assembly; however, WAI2432 contained several large chromosomal rearrangements. These were investigated and confirmed by aligning raw nanopore reads to the *de novo* assembly (Fig. S1B).

### Re-sequencing of *B. sorokiniana* revealed a conserved sub-telomeric region

Chromosome completeness was assessed based on the presence of 5′ and 3′ terminal telomeric repeats. *B. sorokiniana* has a 6 bp telomeric repeat of “AACCCT” extending up to 450 bp, as identified in the CS10 reference genome (18). Whole chromosome alignments of the nine assemblies revealed a conserved sub-telomeric sequence. The region ranged from 500 bp in Chr_05 to >1 kbp in all other chromosomes and had a pairwise sequence identity of 88.2% (Fig. S2). Chr_08, Chr_09, and Chr_12 showed evidence of RIP mutation across the 3′ end of the telomeric region (see 380–1,000 bp in Fig. S2). Excluding these chromosomes from the analysis, the sequence was 96.5% identical across the remaining 11 chromosomes. The sub-telomeric region was identified at multiple chromosome termini of the newly sequenced isolates with >90% pairwise identity. This sub-telomeric sequence appears to be unique to *B. sorokiniana* as it was not identified in the publicly available *P. nodorum* isolate SN15 nor the *P. tritici-repentis* isolate 1C-BFP, two close relatives of *B. sorokiniana*. Similarly, a BLASTn analysis in the NCBI nr database found no significant hits outside of the *B. sorokiniana* genome. As this region was consistently identified adjacent to the telomeres in nearly every chromosome of CS10, it was used as a second marker of chromosome completeness for our nanopore assemblies. We considered nanopore-assembled chromosomes complete if both termini had a minimum of three telomeric repeats (visually identified) or if ≥650 bp of the sub-telomeric region was present at the termini with ≥70% nucleotide identity. Genome assembly size and completeness for each isolate are summarized in Table 1.

### *ToxhAT* is an active transposon within *B. sorokiniana*

**Fig 1:**
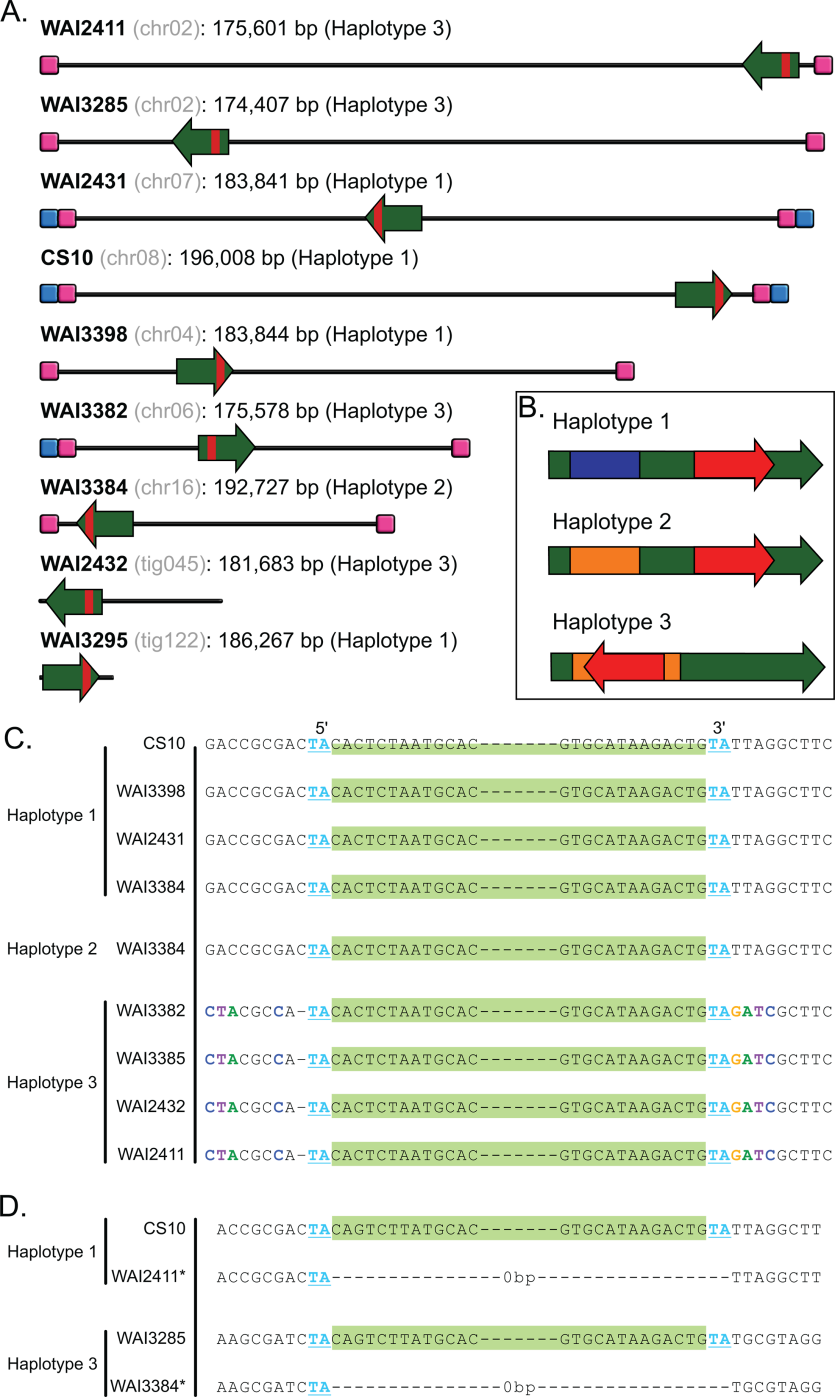
(**A**) Schematic overview of the chromosomal location of *ToxhAT* and the conserved 200 kbp region that is shared between all re-sequenced *B. sorokiniana* isolates. The name of the isolate is shown in bold text with the chromosome or contig name in gray. The exact size of the conserved element is shown in base pairs next to the chromosome name. Chromosomes containing telomeric repeats are indicated with a blue square, and the conserved sub-telomeric sequences are indicated with a pink square. The direction of insertion of the conserved 200 kb is shown by the green arrow. The location of *ToxhAT* is shown by the small red square inside the green arrow. (**B**) A schematic overview of the three haplotypes of the conserved mobile element. *ToxhAT* is shown in red with its orientation indicated by the arrow. (**C**) Alignment of all re-sequenced isolates showing a partial sequence of the TIR of *ToxhAT* in light green. The “TA” target site duplication found on either side of *ToxhAT* in all three configurations is highlighted in bold blue text. (**D**) Alignment of the *ToxhAT* to an empty insertion site in a different isolate. The “TA” TSD only appears once in the empty insertion site, but twice on either side of *ToxhAT*. Color coding indicates the *ToxhAT* TIRs and TSD as in panel C above.

*ToxhAT* was manually annotated in each genome using the CS10 annotation as a reference. The full transposon was present as a single copy in all newly sequenced isolates, with pairwise nucleotide identities of ≥99.5%, when aligned to CS10. All manually annotated genes within *ToxhAT* were present, with pairwise nucleotide identity of ≥98%. Two *ToxA* haplotypes were identified in the newly sequenced isolates, both matched previously documented *ToxA* sequences (14, 23). *ToxhAT* was identified in a different genomic location in all re-sequenced *B. sorokiniana* isolates, mostly in different chromosomes (Fig. 1A). Whole chromosome alignments also identified a conserved ~200 kbp region carrying *ToxhAT* in all nine isolates (Fig. 1A). We confirmed the chromosomal location of the 200 kbp region in each chromosome, including *ToxhAT*, through the alignment of the raw reads to each *de novo* assembly (Fig. S3).

To investigate how this 200 kbp may have translocated or moved between different chromosomes, this region was extracted from each isolate and realigned with all sequences in the 5′–3′ orientation as it is found in CS10 Chr_08. This alignment showed that these 200 kbp blocks had conserved edges. The exact length of the conserved DNA was 174–196 kbp and existed in three distinct haplotypes shown schematically in Fig. 1B (Table 2). Haplotype 1 could be distinguished from the other two by a unique 35 kbp sequence that was not present in other haplotypes. In Haplotypes 2 and 3, this sequence was replaced by a different 30 kbp sequence (Fig. 1B). Excluding these regions that were unique to either Haplotype 1 or Haplotypes 2 and 3, the remaining 160 kbp DNA alignment showed ~91.3% pairwise nucleotide identity. Haplotypes 1 and 2 both carried *ToxhAT* at a shared 3′ position, whereas in Haplotype 3, *ToxhAT* was inverted and translocated to a 5′ position within the larger 200 kbp (Fig. 1B). As *ToxhAT* was present in two distinct locations within the three haplotypes, we investigated the empty insertion sites for evidence of active translocation via a transposase, namely target-site duplications. A multiple sequence alignment of all nine *ToxhAT* sequences from Haplotypes 1 and 2 (3′ position) against Haplotype 3 (5′ position) identified a 2 bp TSD of “TA” bounding the transposon (Fig. 1C). In contrast, the empty insertion sites had only one “TA” present (Fig. 1D). This shows that *ToxhAT* is likely an active transposon with a two-base pair “TA” TSD.

**TABLE 2:** Chromosomal location, length, orientation, and a summary of gene content in each *Sanctuary* haplotype

Isolate	Chromosome	Min (bp)^*a*^	Max (bp)^*b*^	Length (bp)	Orientation^*c*^	*ToxhAT* location^*d*^	Total genes annotated	Haplotype	Average haplotype length (bp)	Average gene count per haplotype
CS10	Chr_08	1,875,320	2,071,325	196,005	+	3′	55	H1	187,479	52
WAI2431	Chr_07	1,731,625	1,915,465	183,840	−	3′	52			
WAI3295	tig122	1	186,228	186,227	+	3′	49			
WAI3398	Chr_04	633,088	816,931	183,843	+	3′	51			
WAI3384	Chr_16	165,966	358,692	192,726	−	3′	57	H2	192,726	57
WAI2411	Chr_02	3,435,149	3,610,749	175,600	−	5′	52	H3	176,816	53
WAI2432	tig045	42,029	223,711	181,682	−	5′	54			
WAI3382	Chr_06	734,719	910,295	175,576	+	5′	52			
WAI3285	Chr_02	1,563,991	1,738,397	174,406	+	5′	52			

^*a*^ Chromosomal position that is the start of the *Starship*.

^*b*^ Chromosomal position that is the end of the *Starship*.

^*c*^ With reference to the orientation of CS10 chromosomes, whether the conserved start of the configuration is on the positive or negative strand.

^*d*^ Location of ToxhAT within the haplotype.

### *ToxhAT* is moving as cargo within a *Starship* transposon in *B. sorokiniana*

After investigating the movement of *ToxhAT* alone, we next sought to investigate the movement of the larger conserved 200 kbp element. Our previous work with *P. tritici-repentis* had already identified a giant *Starship* transposon, *Horizon*, which carries the smaller *ToxhAT* (24)*. Starships* are defined as large transposons, whose first gene, encoded at the 5′ edge, encodes a tyrosine recombinase (YR) transposase (25, 26). This YR-protein contains a Domain of Unknown Function (DUF) 3435 and is referred to as the “Captain” (25). In addition to the YR-captain, *Starships* also contain several other conserved protein families known as “accessory” genes, which include NOD-like receptors (NLRs), Patatin-like phospholipase (PLP) domain-containing proteins, Ferric-reductases (FREs), and DUF3723 proteins. Using this knowledge of *Starship* gene content, each *B. sorokiniana* haplotype was manually annotated, and all genes were assigned a putative function based on conserved domains (Table S2). Excluding genes within *ToxhAT*, 59 unique genes were identified across all three haplotypes (Table 2). Notably, the first gene at the 5′-end in all three configs was predicted to encode a DUF3435 domain. In all three haplotypes, the DUF3435 Captain is found ~350 bp from the 5′ edge, and the average pairwise identity of the gene was 96% across all nine isolates. The gene product is predicted to contain a YR domain at the middle of the translated product and a C-terminal DNA-binding domain (Fig. S4). In addition to this Captain gene, each haplotype contained several predicted “accessory” genes, supporting the classification of this region within *B. sorokiniana* as a *Starship* transposon. All three haplotypes included two putative FREs, three NLRs, and three PLP proteins (Table S2). Alignment of the Captain YR from *P. tritici-repentis* and the previously described *Horizon Starship* showed that these proteins were not closely related; therefore, we named this novel *Starship* in *B. sorokiniana Sanctuary*. More detailed comparisons of the two *Starship*s are presented further below.

### Size variation of *Sanctuary* transposons present** in** *B. sorokiniana*

**Fig 2:**
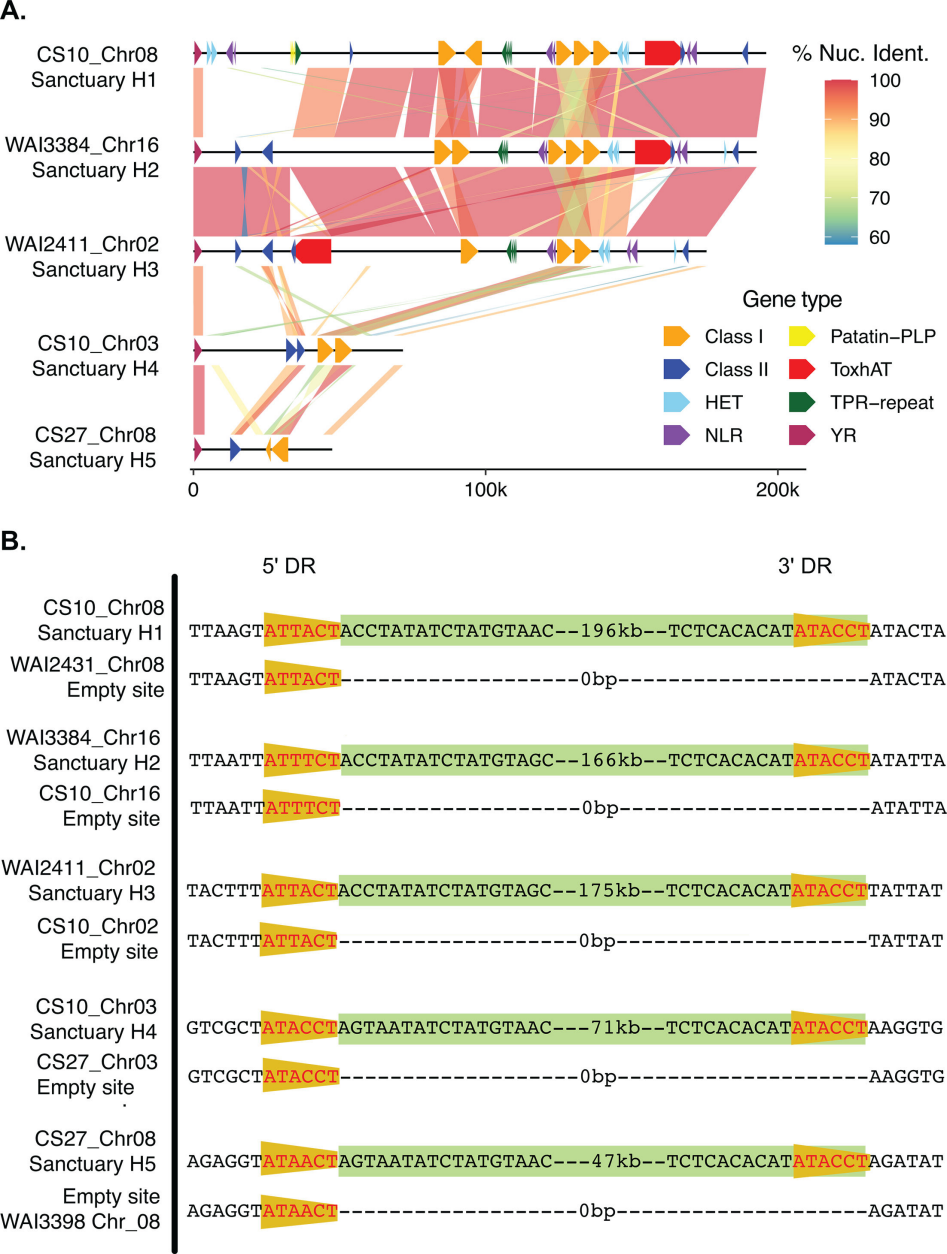
(**A**)Alignment of all distinct *Sanctuary* haplotypes (H1-H5) found in both *ToxA+* and *toxa−* isolates. Notable cargo or accessory genes are drawn as colored arrows, and the gene classification is detailed in the legend. Not all predicted genes are shown to improve the legibility of the figure. (**B**) Alignment of the edges of *Sanctuary* to an “empty” site from the same location in another isolate. Isolate names and chromosomes are given on the far left. The 6 bp DR is shown in the orange triangle, and the truncated *Starship* sequence is highlighted in the green box with its size in base pairs shown. Empty insertion sites are shown with dashes to align the edges.

Using the Captain gene as a search query, we identified two additional *Sanctuary* transposons within *B. sorokiniana* that do not carry *ToxhAT* as cargo. A 72.7 kbp *Sanctuary* was found at the same Chr_03 locus in all nine *ToxA+ B. sorokiniana* genomes. In addition, a 47.5 kbp *Sanctuary* was identified on Chr_08 in *tox*a− isolate CS27. The latter was at a similar, but not identical location as the full-length *Sanctuary* in Chr_08 of isolate CS10. Alignment of each of these smaller *Sanctuaries* with the full-length *Sanctuary* found that the first 3.8 kbp, including the Captain, was conserved with a nucleotide sequence identity of 98.5% (Fig. 2A). The final 3.1 kbp was conserved with a sequence identity of 88.7% that included one annotated gene, *ROU50*. A BLASTp search with the amino acid prediction of this gene showed that it contains a conserved chromodomain (cd00024), which is common in certain retrotransposon families (27). Given the sequence similarity of these *Sanctuary* variants to the larger haplotypes carrying *ToxhAT,* these were given additional haplotype names (Fig. 2A). Outside of these conserved edges, these shorter haplotypes shared little sequence similarity with each other and with *Sanctuary* (<70%). These smaller haplotypes were not identified in any species outside of *B. sorokiniana* via NCBI BLASTn search.

*Starship* transposition has now been experimentally shown to be mediated by the YR-encoding Captain (8). This YR-recombinase-driven excision and re-insertion creates duplications of the target sequence at the insertion site known as direct repeats (DRs) (8). *Starship* DRs appear to be highly variable in length and sequence conservation, though some families do appear to have some specificity (26). As such, the conserved boundaries of *Sanctuary* were analyzed for the presence of DRs. Close examination of the sequence immediately upstream of the 5′-end revealed a 6 bp sequence, which was present in the empty insertion sites of all isolates (Fig. 2B; Fig. S5). This 6 bp sequence was not perfectly conserved but had a consensus sequence of “ATWHCT,” where W is an A or a T, and H is an A, T, or C. All five *Sanctuary* haplotypes showed perfect conservation of the last six base pairs, which we now refer to as the 3′ DR (“ATACCT”) (Fig. S5). This unique multi-strain data set provides evidence that in *B. sorokiniana*, *Sanctuary* excises through a recombination-driven mechanism that potentially carries 3′ DR sequence with the transposon as it moves (8, 28, 29). Attempts to amplify a circular intermediate sequence by PCR from CS10 grown under different environmental conditions were not successful in obtaining a specific amplicon (data not shown). Further optimization of the conditions required to induce *Starship* movement and extract eccDNA is required before this hypothesis can be explored in more detail.

### Sequence comparison **of** *ToxhAT* carrying *Starships***:** *Sanctuary and Horizon*

**Fig 3:**
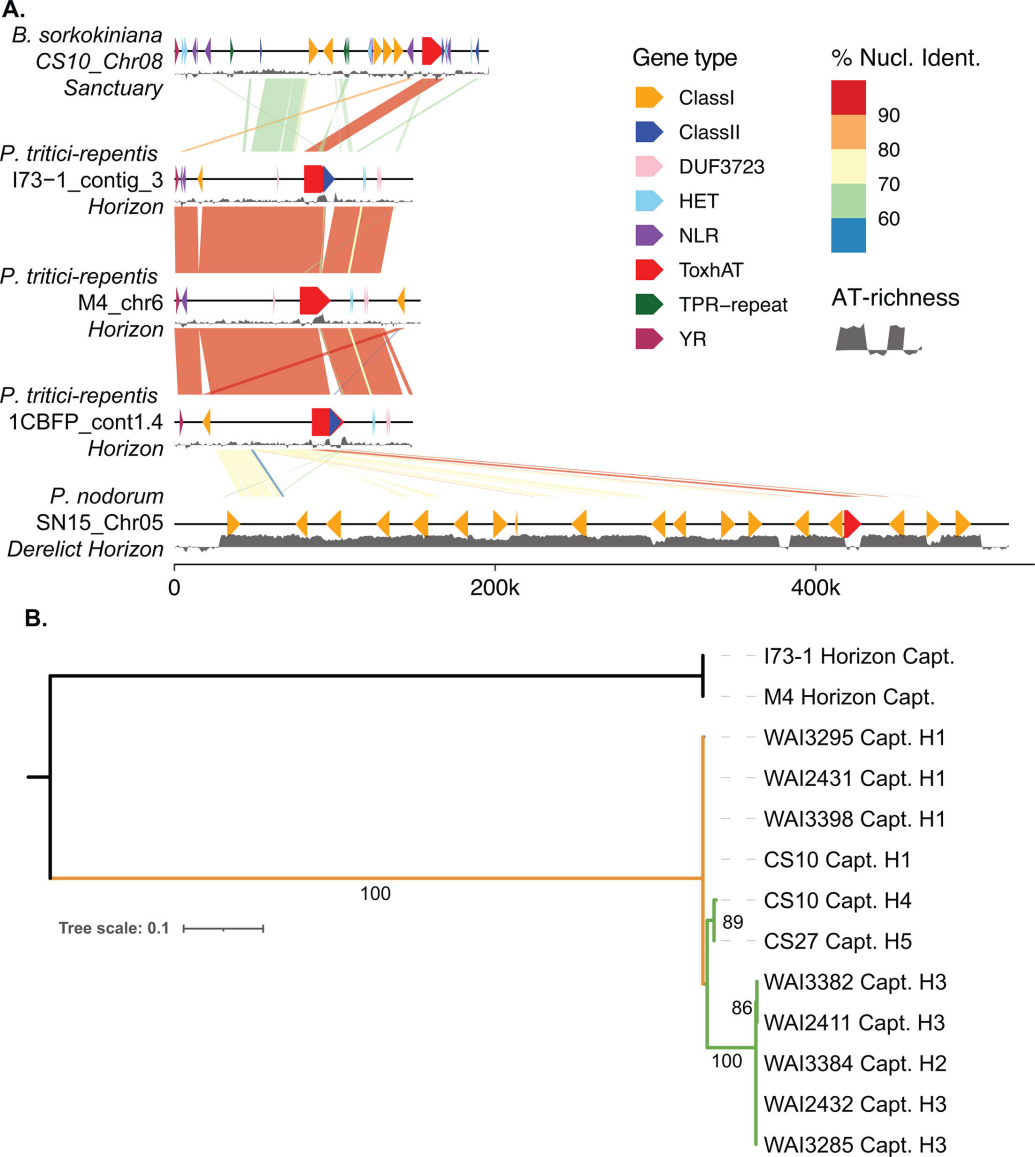
(**A**) Lastz alignment of CS10 *Sanctuary* in *B. sorokiniana* to *Horizon* in *P. tritici-repentis* isolates M4, I73-1, and 1C-BFP and to the fragments of *Horizon* found in *P. nodorum* SN15. Chromosomes are shown as black lines with *Starship*-associated genes plotted on top of the line and colored according to the legend. Links between chromosomes show alignments with the percent nucleotide identity shown according to the legend. Below each chromosome, there is an AT-richness track shown in dark gray ribbons. The AT-richness line is centered on 50% AT, where the ribbon drawn above is >50% AT and the ribbon below is <50%. (**B**) A phylogenetic tree showing the relationships between the *Sanctuary* and *Horizon* captain protein sequences. All *Sanctuary* haplotypes are included, with the name of the isolate and haplotype number indicated. Numbers on the branches indicate bootstrap values, confidence estimated from 1,000 bootstraps by RaxML.

Our discovery of *Sanctuary* in *B. sorokiniana* occurred concurrently with the discovery of another *Starship*, *Horizon,* that was found to harbor *ToxhAT* in *Pyrenophora tritici-repentis* (24). Whole *Starship* alignments between both *Sanctuary* and *Horizon* revealed no regions with high similarity except for *ToxhAT* (Fig. 3A). These alignments offered the opportunity to further examine the shared DNA between *P. tritici-repentis* and *P. nodorum,* which we previously reported to have regions exceeding 50 kb that shared greater than 70% sequence identity between these two species (18). The alignment of *Horizon* in *P. tritici-repentis* to *P. nodorum* showed that small fragments of *Horizon* are present immediately adjacent to *ToxhAT* in both the 5′ and 3′ directions in *P. nodorum* isolate SN15 (Fig. 3A). In between these alignments are several annotated retrotransposons, which have been mutated by RIP as evidenced by the high AT-richness score across this entire region in *P. nodorum* isolate SN15 (Fig. 3A) and reported previously (18). These alignments demonstrate that an identical *ToxhAT* has been captured independently by two different *Starships, Sanctuary* and *Horizon,* and that *Horizon* is present in *P. tritici-repentis* and the derelict *Horizon* is present in *P. nodorum*. This suggests that Horizon was the unit of horizontal transfer between these two species. We next sought to look for homology at the amino acid level for each Captain and again detected little sequence similarity between the two proteins (Fig. 3B). As expected, this phylogenetic analysis shows that the *Sanctuary* Captains are all closely related to each other, forming a highly supported monophyletic group that is distant from the *Horizon* Captains that form their own clade (Fig. 3B).

### Structural predictions of the *Starship* YRs show similarity to the bacteriophage P1 Cre recombinase

**Fig 4:**
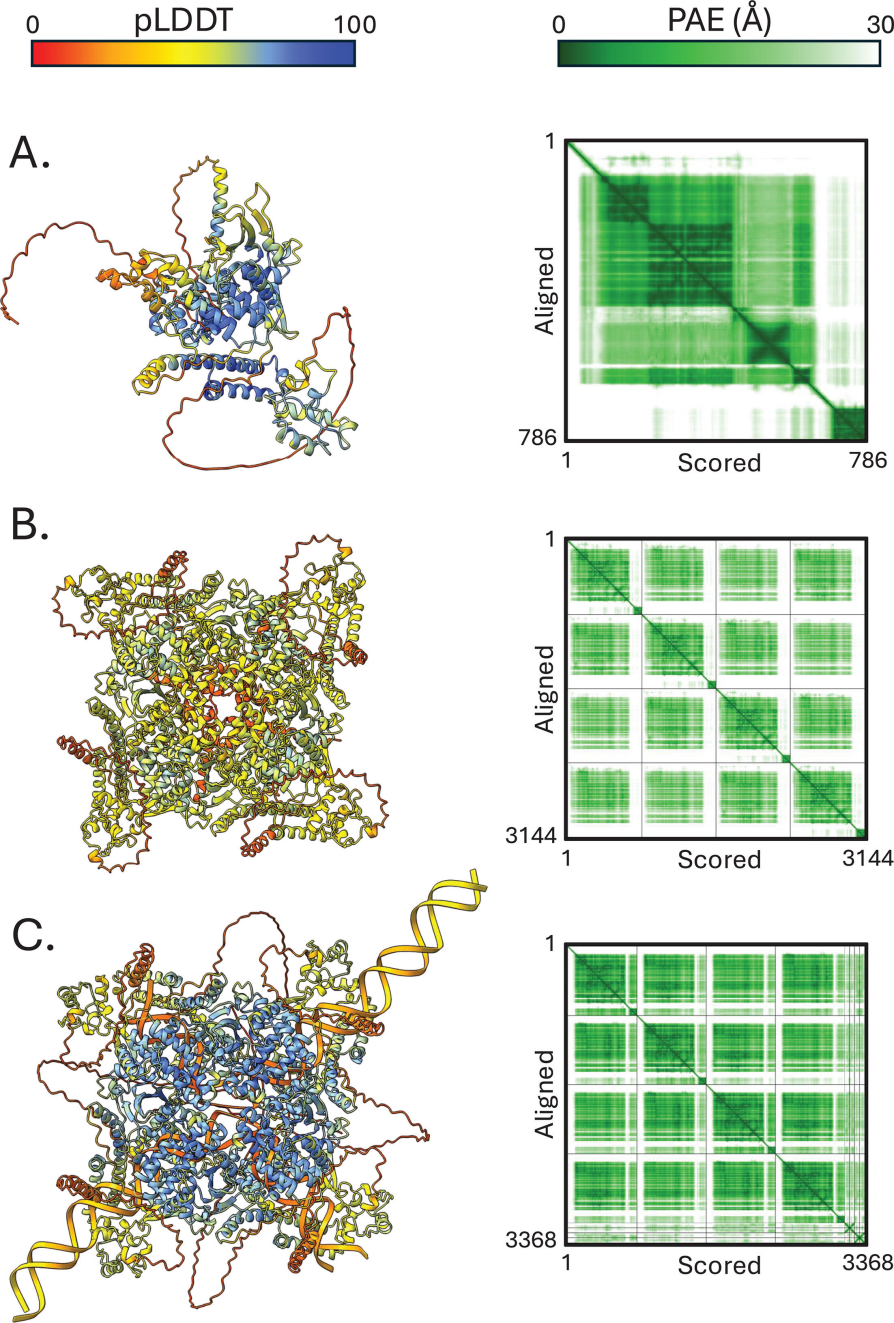
AlphaFold3 predictions of the *Sanctuary* tyrosine recombinase. (**A**) Prediction of the YR monomer (pTM = 0.58). (**B**) Prediction of the YR tetramer (pTM = 0.63 and ipTM = 0.60). (**C**) Prediction of the YR tetramer with 50 bp DNA including the direct repeat regions (pTM = 0.75 and ipTM = 0.73). All models (left) are colored by pLDDT. Predicted aligned error (PAE) plots are shown to the right of each model.

Previous research on the *Starship* transposon *Hephaestus* identified the YR, HhpA, as being structurally similar to the bacteriophage P1 Cre recombinase (8). Consistent with all described *Starship* Captains thus far, the 786 aa Captain from *Sanctuary* was predicted by NCBI BLASTp and Phyre2 protein fold recognition server to contain a site-specific YR domain from 111 to 463 with 98.8% confidence, and a DNA-binding domain from 711 to 784 with 95% confidence (Fig. S4). Using AlphaFold3, we generated a monomeric prediction of the *Sanctuary* YR to determine whether it also shared structural similarity with the P1 Cre recombinase (Fig. 4A) (30). Despite global confidence being at the lower end of what could be considered a confident prediction (pTM = 0.58), predicted local distance difference test (pLDDT) shows some highly confident regions of the protein model (Fig. 4A). Using this model, we carried out a structural homology search via Dali (31). Results indicated that the Sanctuary YR has structural homology to the Tre recombinase and Cre recombinase, similarly shown for the HhpA YR by Urquhart et al. (8). In particular, our results included the P1 Cre recombinase (PDB: 3C29) with a root-mean-square deviation of 3.3 Å across 264 equivalent residues. Due to this similarity, and previous work showing the P1 Cre recombinase assembles into a tetrameric complex (32), we generated tetrameric predictions of *Sanctuary* YR (Fig. 4B) and *Horizon* YR (Fig. S6A). Superimposition of both tetrameric predictions shows a shared global fold between *Sanctuary* and *Horizon* (Fig. S6B). While global confidence of the tetrameric *Sanctuary* YR remained similar to the monomer prediction (pTM = 0.63 and ipTM = 0.60), the previously highly confident regions identified by pLDDT were lost. To rescue the local prediction confidence, we generated a tetrameric YR complex with two double-stranded DNA regions that include DRs from *Sanctuary* (Fig. 4C). Specifically, the input DNA region consisted of the DRs plus 50 bp upstream and downstream of it. This prediction saw an increase in global confidence metrics (pTM = 0.75 and ipTM = 0.73) and an increase in local confidence when measured with pLDDT. This tetrameric model with the dsDNA modeled was taken forward for comparative analysis against the P1 Cre recombinase.

**Fig 5:**
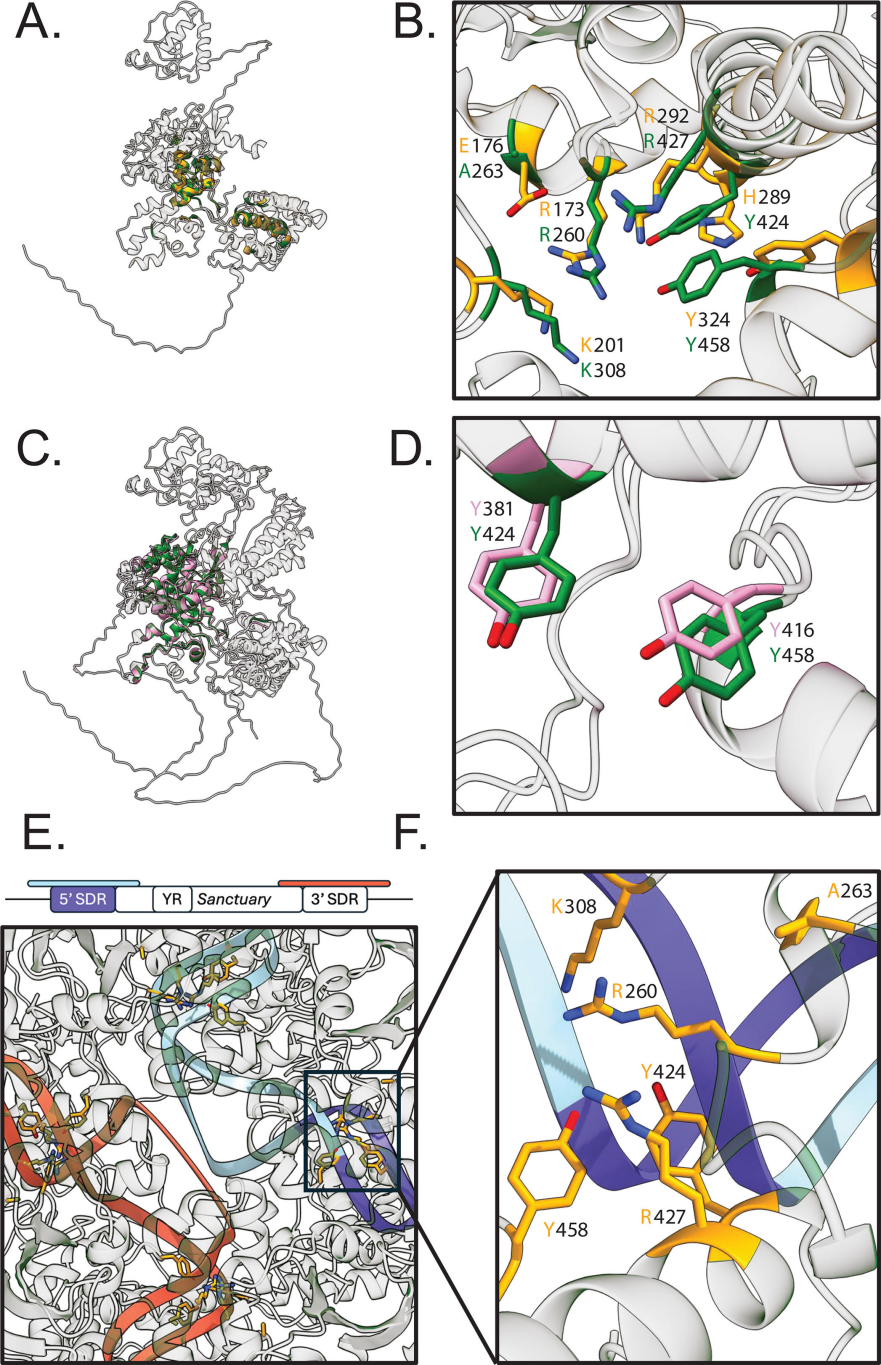
Comparison of *Sanctuary* YR prediction to HhpA and the P1 Cre recombinase. (**A**) Superimposition of the predicted *Sanctuary* YR (green) monomer aligned to the P1 Cre recombinase structure (yellow). Colored regions represent aligned regions. (**B**) Superimposition of the previously identified catalytic residues of the P1 Cre recombinase and their corresponding residues within the *Sanctuary* YR model. (**C**) Superimposition of the predicted *Sanctuary* YR (green) monomer aligned to the HhpA model (pink). Colored regions represent aligned regions. (**D**) Superimposition of the previously identified tyrosine residues (Y381 and Y416) of HhpA required for *Starship* transposition and their corresponding residues (Y424 and Y458) within the *Sanctuary* YR model. (**E**) Overview of the *Sanctuary* YR tetramer modeled with DNA, showing all four putative active sites are located close to DNA. (**F**) Putative active site residues (yellow) of the *Sanctuary* YR showing proximity to the 5′ direct repeat region.

Superimposition of the P1 Cre recombinase tetrameric model (Fig. S7A) with the *Sanctuary* YR tetramer (Fig. S7B) shows a similarity in the global folds (Fig. S7C), demonstrating a similarity including domain arrangement, despite the *Sanctuary* YR prediction being larger. However, when comparing monomers, there is little structural alignment (Fig. 5A). The P1 Cre recombinase uses a large basic surface to bind DNA, with the active residues (R173, E176, K201, H289, R292, W315, and Y324) residing at the interface with the DNA molecule (Fig. 5B) (32). Despite this, the region that does structurally align well includes most of the previously described P1 Cre recombinase catalytic residues. Using this alignment, we identified the corresponding residues within the *Sanctuary* YR (Fig. 5B). These corresponding residues are R260 (R173), A263 (E176), K308 (K201), Y424 (H289), R427 (R292), and Y458 (Y324). While both E176 and H289 have structurally homologous counterparts in the *Sanctuary* YR, they are different residues. Furthermore, the Y424 and Y458 residues identified within the *Sanctuary* YR are part of a small structurally conserved region with HhpA (Fig. 5C). Their corresponding residues within HhpA, Y381 and Y416 (Fig. 5D), have been previously reported to be necessary for YR function within *Hephaestus* (8). This active site is located close to the DR DNA within all four subunits of the tetramer prediction (Fig. 5E). Furthermore, one of the putative active sites is found at the 5′ DR sequence; however, the same is not observed with the 3′ DR (Fig. 5E). Taken together, we hypothesize a putative active site of the *Sanctuary* YR consisting of R260, A263, K308, Y424, R427, and Y458 (Fig. 5F). Attempts to predict interactions between the DNA and the putative active site residues were made; however, none could be made with sufficient confidence.

## DISCUSSION

This study presents new insights into the previously discovered class II *ToxhAT* transposon that carries the agronomically significant *ToxA* effector. Herein, we provide evidence that *ToxhAT* is an active transposon within *B. sorokiniana* based on the identification of a 2 bp “TA” TSD. In addition, we have found that in *B. sorokiniana, ToxhAT* is a “cargo” within a much larger *Starship* transposon, now named *Sanctuary*. There are multiple haplotypes of *Sanctuary* found within the *B. sorokiniana* genomes sequenced thus far, including *Sanctuaries* present in both *ToxA+* and *tox*a− isolates. A comparative analysis of the larger HGT event between all three fungal species known to carry *ToxhAT* shows that this transposon has been acquired independently by two distantly related *Starship* transposons, *Sanctuary* in *B. sorokiniana* and *Horizon* in *P. tritici-repentis* (24). Moreover, this work expands on the previous finding that *P. tritici-repentis* and *P. nodorum* share large sections of homologous DNA outside of *ToxhAT* (18, 24). Whole chromosome alignments now more clearly show that this DNA in *P. nodorum* is a fragmented *Horizon,* which adds to the rapidly growing body of evidence supporting *Starship* transposons as the drivers of horizontal gene transfer between fungal species (33).

With only one TSD present within empty insertion sites, but two flanking *ToxhAT* sequences, we conclude that *ToxhAT* is mobile within all sequenced *B. sorokiniana* isolates. However, the discovery of the “TA” TSD sequence brings the classification of *ToxhAT* into question. A *hAT*-like transposase was reported when *ToxhAT* was initially discovered in *P. nodorum* (13). Typically, *hAT*-type transposons have 8 bp TSD and TIR sequences that range in size from 5 to 27 bp (34). This contrasts with *ToxhAT* having the 2 bp TSD discovered in this study and 74 bp TIR sequences reported previously (18). These characteristics suggest that this transposon has been misclassified. The 2 bp TSD and 74 bp TIR of *ToxhAT* are representative of the *Tc1/Mariner* type of class II transposons. The *Tc1/Mariner* superfamily is characterized by having the same 2 bp “TA” TSD (35) as *ToxhAT*. While typical *Tc1/Mariner* transposons have short TIRs between 20 and 30 bp (36), some have been identified with TIRs up to 169 bp (37). Our earlier work identified a complete *Tc1* transposon (inclusive TIRs) within *ToxhAT* in *B. sorokiniana*. In *P. tritici-repentis,* this site was occupied by a different *Tc1* transposon, and in *P. nodorum,* an LTR retrotransposon was identified. This led us to conclude that these transposons all represented independent nesting events within *ToxhAT* (18). Without further functional studies, we cannot conclude whether the *Tc1* transposase in *B. sorokiniana* is responsible for mobilizing *ToxhAT,* or alternatively, if there is another transposase encoded elsewhere in the genome that facilitates movement. Together, these data suggest that *ToxhAT* is likely a non-autonomous *Tc1/Mariner* transposon that relies on the expression of other transposases within the genome to move. To reflect this more precise description of the target site and acknowledging the uncertainty in classifying this transposon into a superfamily, we propose that *ToxhAT* should be renamed to *ToxTA*.

In all nine *ToxTA*-positive *B. sorokiniaina* isolates, *ToxTA* was embedded within a conserved ~200 kbp region. The region, designated *Sanctuary*, is a *Starship* transposon. *Starships* are a novel group of transposons characterized by their large size, a DUF3435-containing YR as the first gene in the 5′ direction, and for having a cargo and accessory gene structure (25). Cargo genes vary between elements but generally confer fitness advantages to the fungal host in specific environments, while accessory genes fall into several conserved gene families, all of which are found within *Sanctuary* (25, 38). *Sanctuary* has five distinct haplotypes within *B. sorokiniana* with the same 6 bp “ATWHCT” DR sequence and phylogenetic support from the Captain-YR genes showing a close phylogenetic relation. *Sanctuary* is the second identified *Starship* carrying *ToxTA*, the first being *Horizon* in *P. tritici-repentis* and *P. nodorum* (24). While both *Sanctuary* and *Horizon* carry *ToxTA* outside of this region, there is no sequence similarity between them. This indicates that *ToxTA* has been acquired twice, independently, by *Horizon* and *Sanctuary*. Even more recently, *ToxTA* has been identified in a fourth fungal species, *Alternaria ventricosa,* collected from pear (*Pyrus × bretschneideri*), in a putative third *Starship* designated *Frontier* (39, 40). This finding is based on NCBI data alone and does not yet include PCR verification; however, the genome was published as a holotype for this species, indicating that the isolate is a pure culture (39). The process that drives the acquisition of cargo genes, such as *ToxTA,* into *Starships* remains uncharacterized. Moreover, it is still unclear if *ToxTA* is a transposon that is parasitizing the larger *Starships* or if the *Starships* require these “cargo” genes to persist in pathogen genomes (8, 25).

Within *B. sorokiniana, P. nodorum,* and *P. tritici-repentis,* the fitness benefits of carrying the *ToxA* gene are well established (12, 14, 23). However, in all three of these species, *ToxA* is known to exist as a presence-absence polymorphism (13, 14, 41). An exciting future avenue to explore in these existing fungal collections is if the presence/absence polymorphism of *ToxA* is driven by loss of *ToxTA* or the entire *Starship*. This work has already provided some evidence from *B. sorokiniana* that the smaller *Sanctuary* haplotypes that do not carry *ToxTA* or other accessory gene families are in a fixed genomic location (H4 in Chr_03). These haplotypes share a highly identical *Sanctuary* captain and the same 6 bp DR as the largest haplotypes, which supports their classification within the same family and navis (26). However, in the only non-*ToxTA* isolate sequenced thus far, CS27 (original isolate name BRIP27492a), the location of *Sanctuary* H5 was in the tail of Chr_08, not in Chr_03 as observed in the other isolates. Both H4 and H5 share an identical amino acid sequence for the Captain-YR gene, which indicates that mobility of these smaller transposons is possible and fits with experimental evidence showing that the minimal unit of transposition requires only the *Captain* gene (8). The finding of five distinct haplotypes of *Sanctuary* within these 11 *B. sorokiniana* genomes also gives some indication that this *Starship* has been present in the species long enough to accumulate natural sequence variants. Sequencing larger collections of *B. sorokiniana* would provide an opportunity to track the evolutionary history of *Sanctuary* at the population level and potentially reveal further *Sanctuary* haplotypes that either show the decay of *Sanctuary* through time and/or full-length *Starships* that do not carry *ToxTA*. Despite this variation, the current set of *B. sorokiniana* isolates also demonstrates the persistence of the full-length *Sanctuary* through time. For example, isolate CS10 (original name BRIP10943a) is the oldest isolate sequenced thus far and was collected in 1966 in Queensland, Australia. This isolate shares a near-identical sequence with the other three H1-carrying isolates, collected 50 years later in two different Australian states (WAI2431: collected in 2015, WAI3295: collected in 2016, and WAI329: collected in 2017). These population-level studies could provide important clues as to how *Starships* gain and lose genes in their natural fungal host, which remains a key open question in *Starship* evolution (42).

Another remaining question is what mechanism drives *Starship* movement within a genome. Urquart et al. (8) have already shown that mobilized mini-*Hephaestus* elements leave behind a “clean excision” after moving to a new genomic location, which provides strong support that *Starships* transpose within a genome using a site-directed recombination event. Several groups have identified core catalytic residues that are found in other YR-domain containing enzymes, including P1 Cre and Crypton transposases (8, 25, 43). These site-directed recombinase enzymes bind DRs to facilitate a recombination event that results in one circular molecule containing one copy of the DR, while the remaining DR stays in the linear chromosome. Despite this strong evidence for movement via a site-directed recombination event, no work to date has reported data to show the presence of the proposed extra-chromosomal circular intermediate. Therefore, the form in which *Starships* transpose remains an interesting open question (28).

Using AlphaFold3 modeling, we investigated the similarity between the *Sanctuary* and *Horizon* Captains with YRs with previously identified core catalytic residues. Earlier work by Urquhart et al. (8) used AlphaFold to model a monomer of the *Starship* Captains, despite the P1 Cre recombinase being known to function as a homotetramer (32). We sought to expand on this earlier work by modeling both the *Sanctuary* and *Horizon* Captains as homotetramers, which showed distinct global similarity between the Captains and the P1 Cre recombinase. Furthermore, the catalytic residues identified in the P1 Cre recombinase and the *Starship* Captain HhpA were also identified in the *Sanctuary* Captain. The models presented here also identified several additional amino acid residues not previously identified as part of the active site, which can be validated in future functional experiments. P1 Cre recombinase binds a conserved 34 bp *loxP* site and is one of the best-studied site-specific recombinases (32). By predicting the *Sanctuary* Captain homotetramer with DNA regions from the upstream and downstream cut sites, we can predict possible DNA-protein interactions using the P1 recombinase structure as a guide. Interestingly, both the global and local confidence scores for the tetrameric model increased when the SDR and flanking DNA were incorporated, and the model predicts one of the active sites (composed of corresponding residues from the previously identified residues in P1 Cre and HhpA) to be in close proximity to the 5′ SDR of the Sanctuary transposon. Together, these results highlight the growing power of structural bioinformatics to assist in the identification of key catalytic residues without the need to perform large-scale alignments, which are technically difficult with more distantly related proteins.

There is a growing body of literature that now supports multiple independent horizontal transfer events of *Starship* transposons that can, upon transfer, confer fitness benefits to their new fungal hosts (33, 42). Unlike other transposons, it has been argued that horizontal movement of *Starships* is required for them to persist over evolutionary time scales (8, 42). Under this hypothesis, cargo genes are essential for the survival of *Starships* and therefore confer fitness benefits to the fungal host when present. What remains less clear is the role and function of accessory genes within *Starships*. Examples of accessory genes include PLPs and NLRs, some of which have links to self/non-self-recognition pathways in filamentous fungi and have been proposed to be involved or required for horizontal movement (8, 44, 45). *Sanctuary* contains many accessory genes with these domains and is now one of a growing number of *Starships* where there is evidence of active transposition in natural isolates. The conserved chromo-domain-containing protein found in all *Sanctuary* haplotypes as the final gene in the *Starship* is also intriguing, given the role that these domains play in targeting particular families of retrotransposons to methylated tails of heterochromatin (27). There is currently no chromatin state data available for these *B. sorokiniana* isolates, but it is possible that conservation of this gene could help *Sanctuary* target heterochromatin. However, this remains a purely speculative hypothesis until further functional work can be done to establish an experimentally tractable *Sanctuary* within *B. sorokiniana*. Establishing these experimental strains in *B. sorokiniana* and the other *ToxTA*-carrying species is ongoing, which will provide the biological tools required to further explore *Starships* excision, integration, and trafficking through the fungal mycelium from one species to another. Given the mobility and diversity of *Sanctuary* and *ToxTA* in the small number of fungal isolates sequenced thus far, combined with the known fitness benefits of *ToxA,* this is an exciting biological system to further understand transposon-mediated adaptive evolution.

## MATERIALS AND METHODS

### Fungal culture and DNA extraction

*B. sorokiniana* isolates were obtained from the NSW Department of Primary Industries, Wagga Wagga Agricultural Institute (WAI). These isolates and details pertaining to the year and site of collection are provided in Table 1. Fungal cultures were grown on V8-PDA media at 22°C under a 12-h light/dark cycle. Cultures ranging in age from 5 to 10 days were scraped from the agar surface using a sterile razor blade. Harvested cultures were lyophilized for 48 h, and high molecular weight (HMW) DNA was extracted using a modified method from Fulten et al. (46), and our full protocol is available at dx.doi.org/10.17504/protocols.io.k6qczdw.

### Genome sequencing, *de novo* assembly, and scaffolding

Genome assemblies for isolates BRIP10943a (CS10) and BRIP27492a (CS27) were obtained from McDonald et al. (18). All new raw data generated for this study were deposited in NCBI BioProject ID PRJNA1017791. HMW DNA for each isolate was sequenced using the Oxford Nanopore MinIon R9.4 Flow cells with the 1D library kit SQK-LSK08 (47). All DNA samples were cleaned three times using Agencourt AMPure beads prior to starting the 1D library kit (Beckman Coulter, Inc., CA, USA). Genomes were assembled with Canu version 1.6, with a minimum read length of 5 kbp (48). *De novo* genome assemblies were corrected using the trimmed reads output from Canu. Trimmed reads were mapped to the genome with Minimap2 version 2.17 (49), followed by consensus calling with Racon (50). The output consensus sequence from Racon was used as input for iterative correction five times.

Contigs in the polished Nanopore assemblies were assigned chromosome numbers based on the numbering and orientation of the CS10 reference genome (18). The CS10 assembly has 16 complete chromosomes labeled by descending size from chromosome 1 to 16. Six partial contigs were also present, named by descending size, contigs 17–22 (18). LastZ version 1.04.03, implemented in Geneious, was used to align assembled scaffolds to the CS10 reference genome (51). A contig was assigned a chromosome number if >90% of its length aligned to a single CS10 chromosome. In instances where multiple smaller contigs aligned in tandem to form a single CS10 chromosome, the contigs were scaffolded together. A summary of the number of contigs, genome size, presence of telomeres, and final chromosome names for each isolate is provided in Table S1.

### Assessing assembly accuracy and completeness

The quality of each polished assembly was assessed using BUSCO version 4.1.4, run with the Dothideomycetes data set of 3,786 genes (21). BUSCO was run on the command line: busco -m genome -l dothideomycetes_odb10 --cpu 12 -i < isolate.fasta> -o < output_directory>. QUAST was used to compute the percentage of the CS10 genome assembly captured within each final Nanopore assembly (22). The online QUAST interface was used, accessible at https://github.com/ablab/quast. No contig size was excluded.

### Genome annotation

Funannotate version 1.7.4 was used to annotate whole genomes of all WAI isolates and the CS10 genome (https://funannotate.readthedocs.io/en/latest/). Briefly, all assemblies were cleaned using the *funannotate sort* and funannotate mask commands. The funannotate train command was then used to train the pipeline for each isolate using the cleaned assembly file and CS10 RNA-seq data (18). Finally, the funannotate predict command was used to annotate each isolate assembly using the trained pipelines. The maximum intron length was set to 1,000 bp for both training and prediction, and the minimum gene size was kept at the default value of 50 bp. The output of the seven individual gene programs used during the funannotate process is combined into one consensus file with Evidence Modeler (EVM). Funannotate uses “weighting” scores to instruct EVM on which output to accept when two software have conflicting annotations (52). The final weightings were kept to default except CodingQuary (default 2, adjusted 6), and Program to Assemble Spliced Alignments (default 6, adjusted 4) (52). No iteration of the funannotate pipeline was able to annotate the genes encompassed by the *ToxTA* transposon. *ToxTA* was therefore manually annotated in each genome using the CS10 annotation as a reference.

### Putative gene function identification

Putative gene functions were identified using the ExPASy translation tool (https://web.expasy.org/translate/) and analyzed for conserved domains using the online NCBI Conserved Domain Search tool (https://www.ncbi.nlm.nih.gov/Structure/cdd/wrpsb.cgi) and Phyre2 version 2.0 (53). All searches used the Conserved Domain Database, a superset containing NCBI-curated domains, and the Pfam, SMART, COG, PRK, and TIGRFAM databases (54). The *e*-value threshold was set at max 0.01. Phyre2 was run in normal mode, and hits with below 90% confidence in the model were excluded from analysis. Cut-offs for amino acid percentage identity were considered on a case-by-case basis. Hypothetical gene functions were assigned if a hit with a low *e*-value was provided by at least one of these three protein predictors.

### *Starship* alignment and phylogenetic analysis

Whole chromosomes containing *Starships* were aligned using lastz version 1.04.22, and these alignments were plotted using the R package gggenomes (51, 55). The full code and data files used to draw each figure containing an alignment are available at https://github.com/megancamilla/Sanctuary_manuscript/tree/main. Amino acid and nucleotide alignments were conducted with MAFFT version 7.490, and phylogenetic trees were constructed with RaxMLv8 as implemented in Geneious Prime (56, 57). For the phylogenetic tree of the YR Captain gene, the maximum likelihood tree was constructed using the rapid bootstrapping algorithm with the GTR + GAMMA amino acid substitution model and 1,000 bootstrap replicates.

### AlphaFold predictions and structural analyses

All predictions of the *Sanctuary* Captain were generated via AlphaFold3. The Dali protein structure comparison server was used to compare the *Sanctuary* monomer to existing protein structures within the Protein Data Bank (31). Prior to Dali, the output mmCIF file from AlphaFold3 was converted to PDB format via Gemmi. Structural analyses were performed and visualized with ChimeraX (58).

## Data Availability

All data are available under NCBI project numbers PRJNA505097 and PRJNA1017791. New BioSamples created for this paper are SAMN39994716–SAMN39994722. New Nanopore raw sequence data are in NCBI’s SRA accessions SRR28048243–SRR28048237. Full genome assemblies and annotations, including detailed annotations of each *Sanctuary Starship*, are available at https://github.com/megancamilla/Sanctuary_manuscript.

## SUPPLEMENTAL MATERIAL

Supplemental Figures mbio.01371-25-s0001.pdf Figures S1 to S7.

Table S1 mbio.01371-25-s0002.xlsx Genome summary statistics for newly sequenced *B. sorokiniana*.

Table S2 mbio.01371-25-s0003.xlsx Detailed functional descriptions of each *Sanctuary* gene.

ASM does not own the copyrights to Supplemental Material that may be linked to, or accessed through, an article. The authors have granted ASM a non-exclusive, world-wide license to publish the Supplemental Material files. Please contact the corresponding author directly for reuse.
