# Supplementary material for: Sanctuary: a Starship transposon facilitating the movement of the virulence factor ToxA in fungal wheat pathogens
Source: mBio. 2025 Sep 4;16(10):e01371-25. doi: 10.1128/mbio.01371-25 (PMC12506042; doi:10.1128/mbio.01371-25)

Supplementary Figure 1. A) Raw nanopore reads (light green) aligned against isolate WAI3382 Chr12_tig17. The region of the chromosome highlighted in red shows the location of rDNA repeats. The black dashed line shows a slope y=1, where by every base in the read would match a base in the chromosome it is aligned against. **B)** Raw nanopore reads from isolate WAI2432 aligned against tig001 from the *de novo* assembly. The region in red highlights the point at which tig001 of WAI2432 transitions from chr07 to chr05 of CS10. Here the raw reads support the *de novo* assembly that tig001 in isolate WAI2432 is a fusion of chr05 and chr07 from isolate CS10.

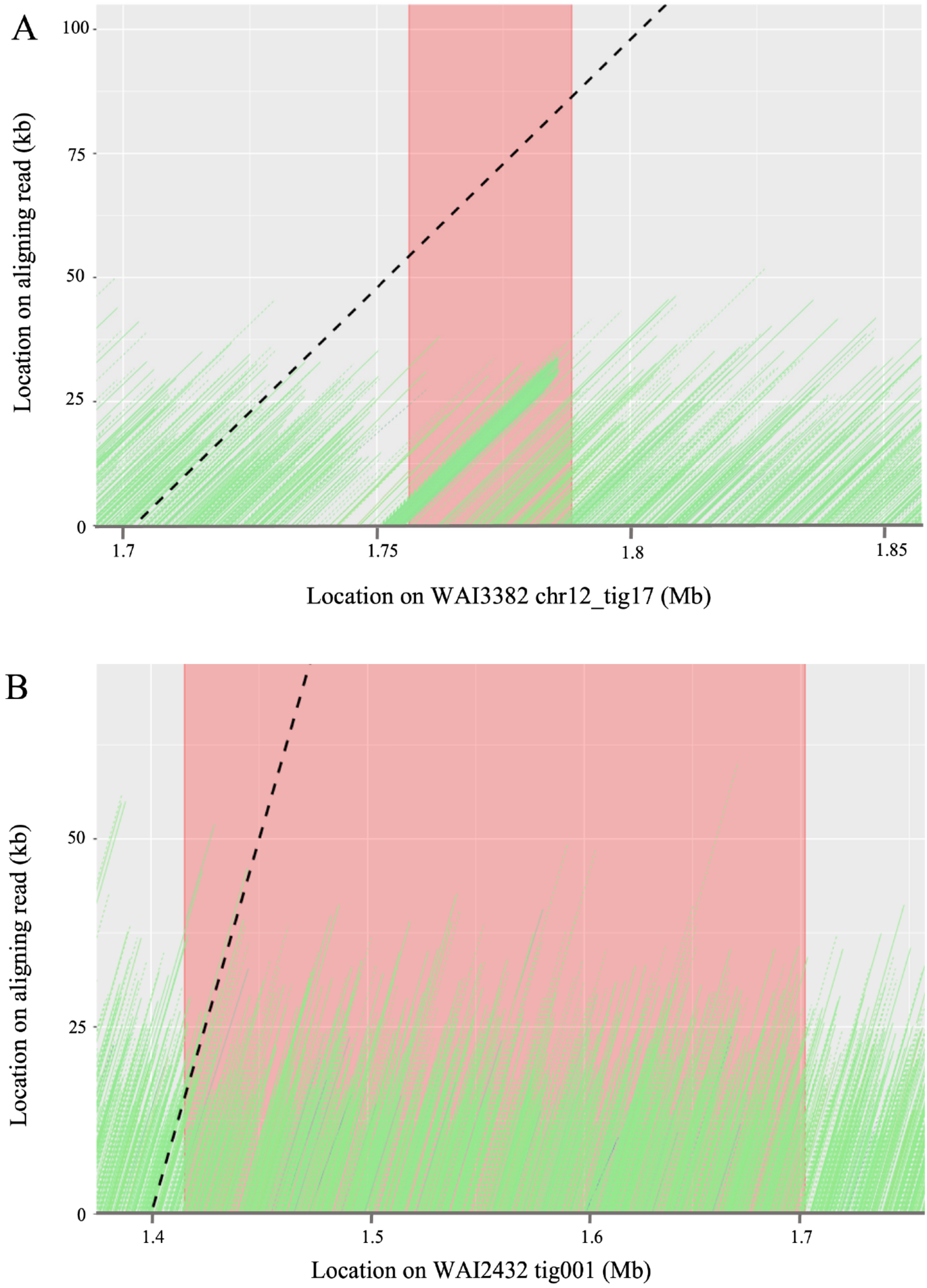

Supplementary Figure 2. Alignment of the conserved sub-telomeric region from CS10. The alignment of the first 962bp of each CS10 chromosome that contains the subtelomeric region. The alignment contains one whole telomeric repeat from bases 4-8. Aligned using MAFFT aligning tool. Coloured bases indicate a deviation from the consensus sequence. An identical 307bp segment was identified in CS10 Chr07 (starting base 210) and WAI3285 Chr06 (starting base 864). This region was removed from this alignment for ease of reading but each deletions has been highlighted light blue. The subtelomeric region in chr05 was significantly shorter (625bp) and was truncated at the point it stops aligning

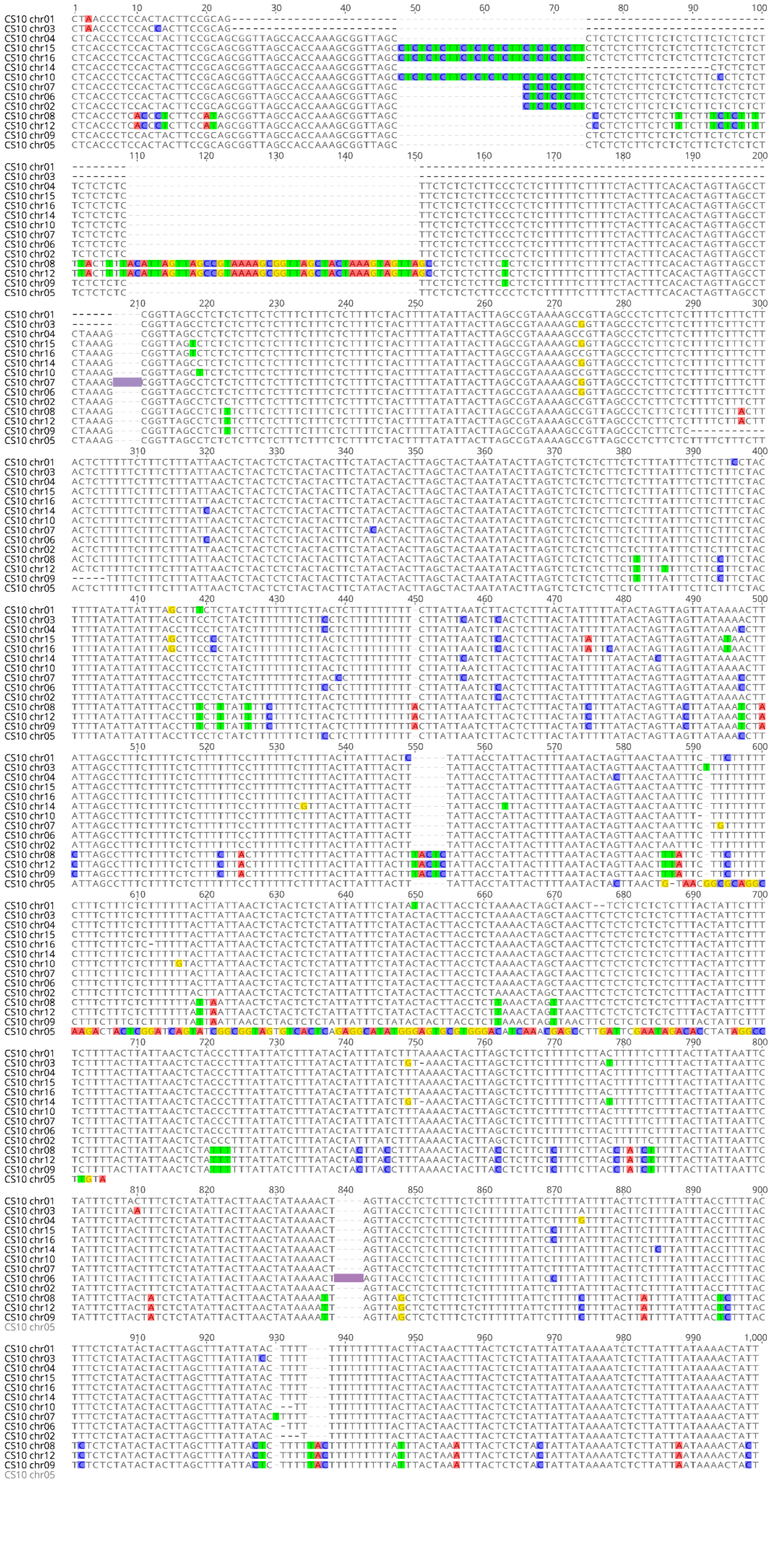

.

Supplementary Figure 3. Alignments of raw reads against the contigs/chromosomes on which the moving segments were located. Large purple shaded region indicated the boundaries of each putative starship, whilst the smaller, red section indicates the location *ToxhAT*. A) Sanctuary haplotype1 and haplotype 2 isolates and B) Haplotype 3 isolates. The small green arrow in the top right corner indicates the *Starship* haplotype number and orientation. Isolate WA3295 is not shown because this region was a single contig that did not assemble into a chromosome.

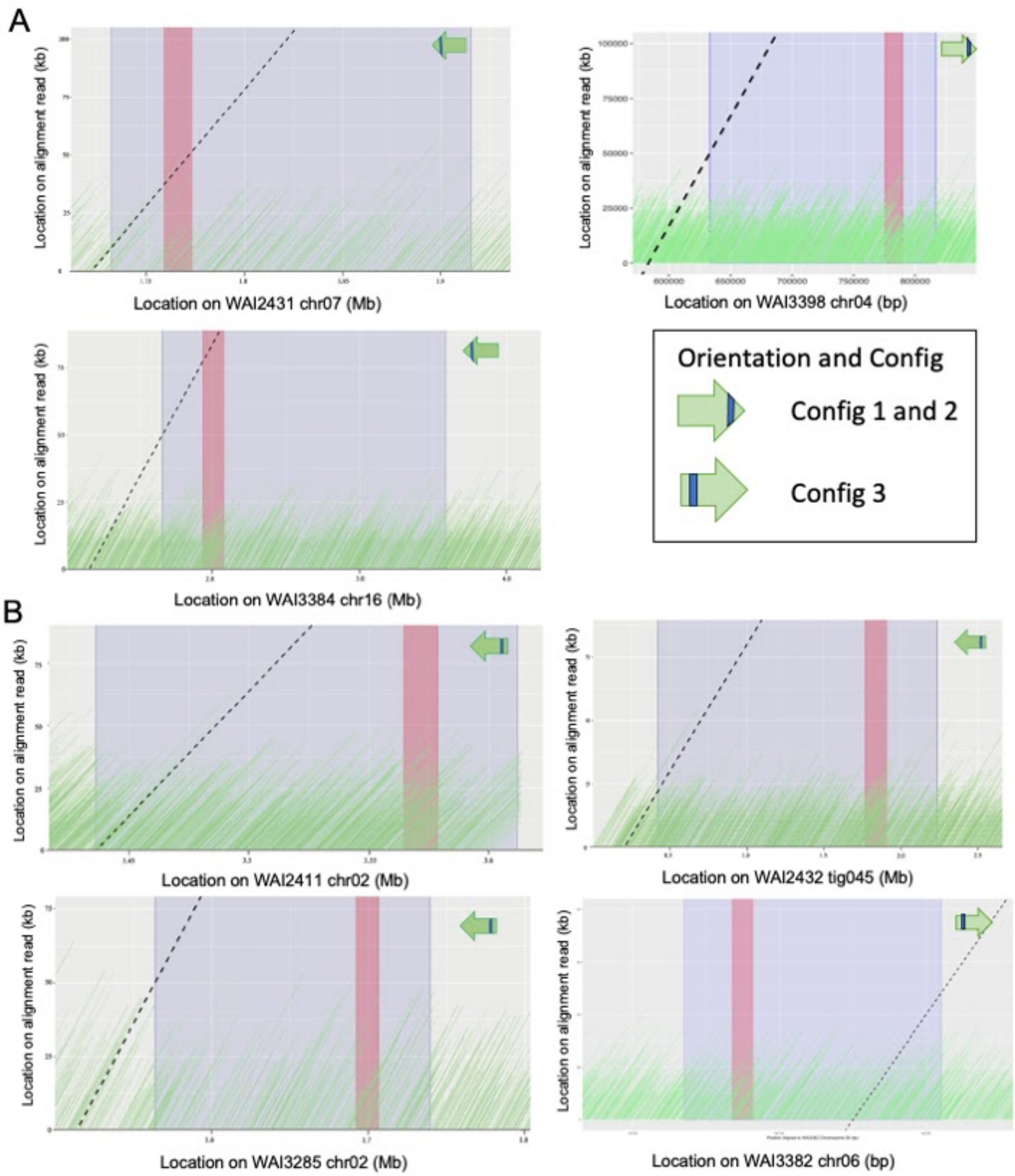

Supplementary Figure 4. Schematic overview of the DUF3435 encoding Captain from Sanctuary. The DUF3435 domain is highlighted from amino acid positions 221-616 and the putative catalytic tetrad is highlighted in light blue. A second predicted Zinc-finger C2H2 DNA binding domain (759-782) is shown in light green.

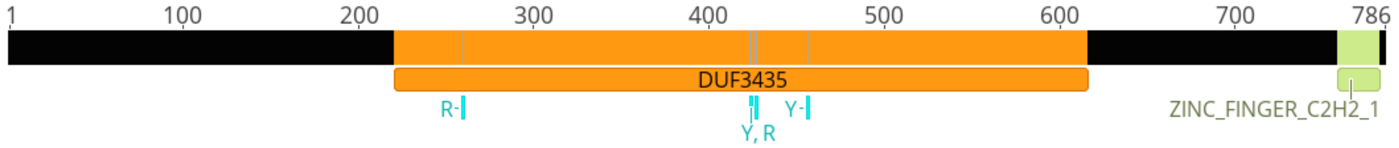

Supplementary Figure 5. Alignment of the edges of *Sanctuary* to an “empty” site from the same location in another isolate. Isolate names and chromosomes are given on the far left, the 6- bp SDR is shown in the orange triangle and the truncated *Starship* sequence is highlighted in the green box with its size in bp shown. Empty insertion sites are shown with dashes to align the edges.

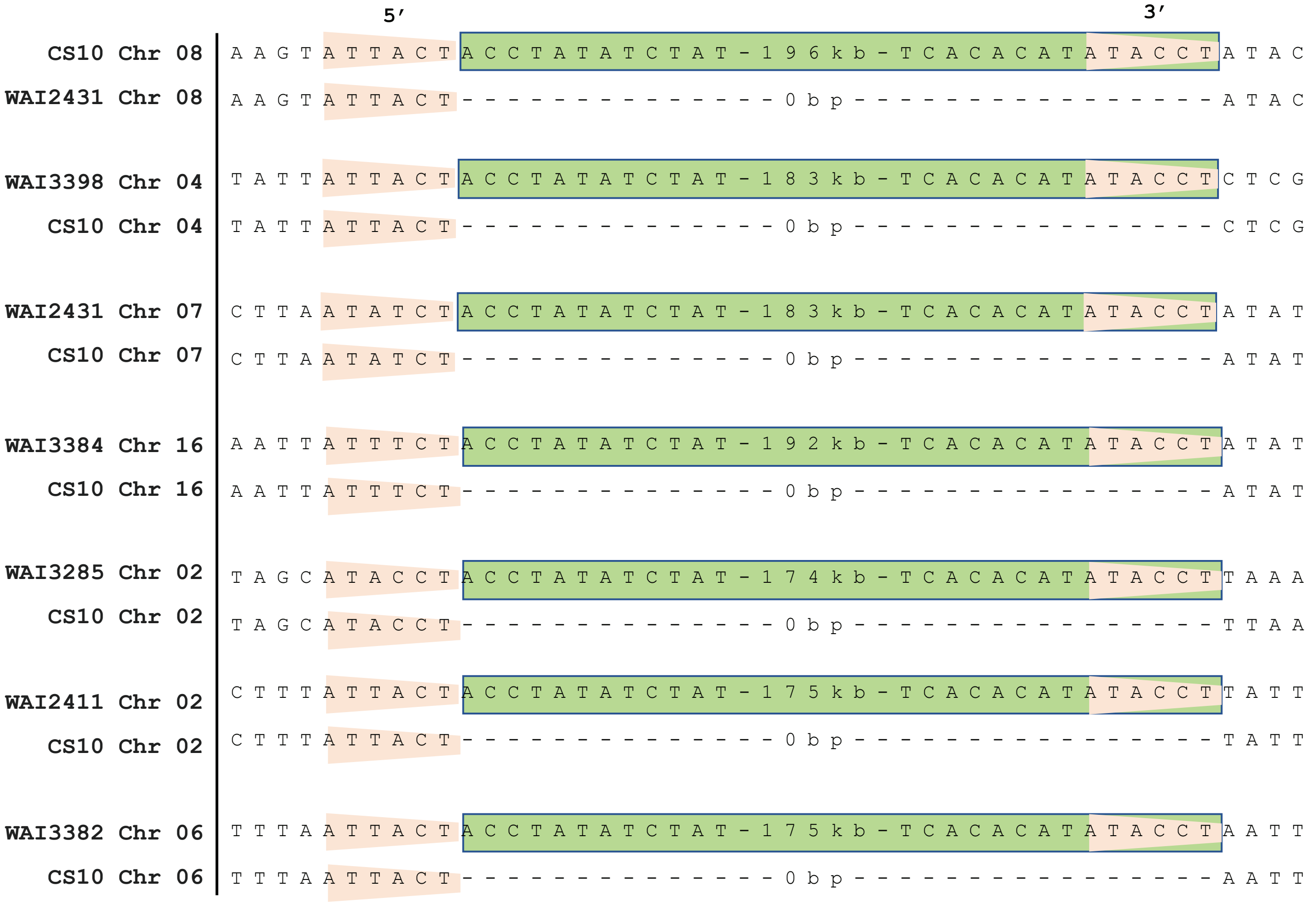

Supplementary Figure 6: prediction of the tyrosine recombinase (YR) from *Horizon*. A) AlphaFold3 prediction of the *Horizon* YR tetramer coloured by predicted local distance different test (pLDDT) (left) and predicted aligned error (PAE) plot (right). B) Superimposition of the *Sanctuary* YR tetramer (green) with the *Horizon* YR tetramer (purple).

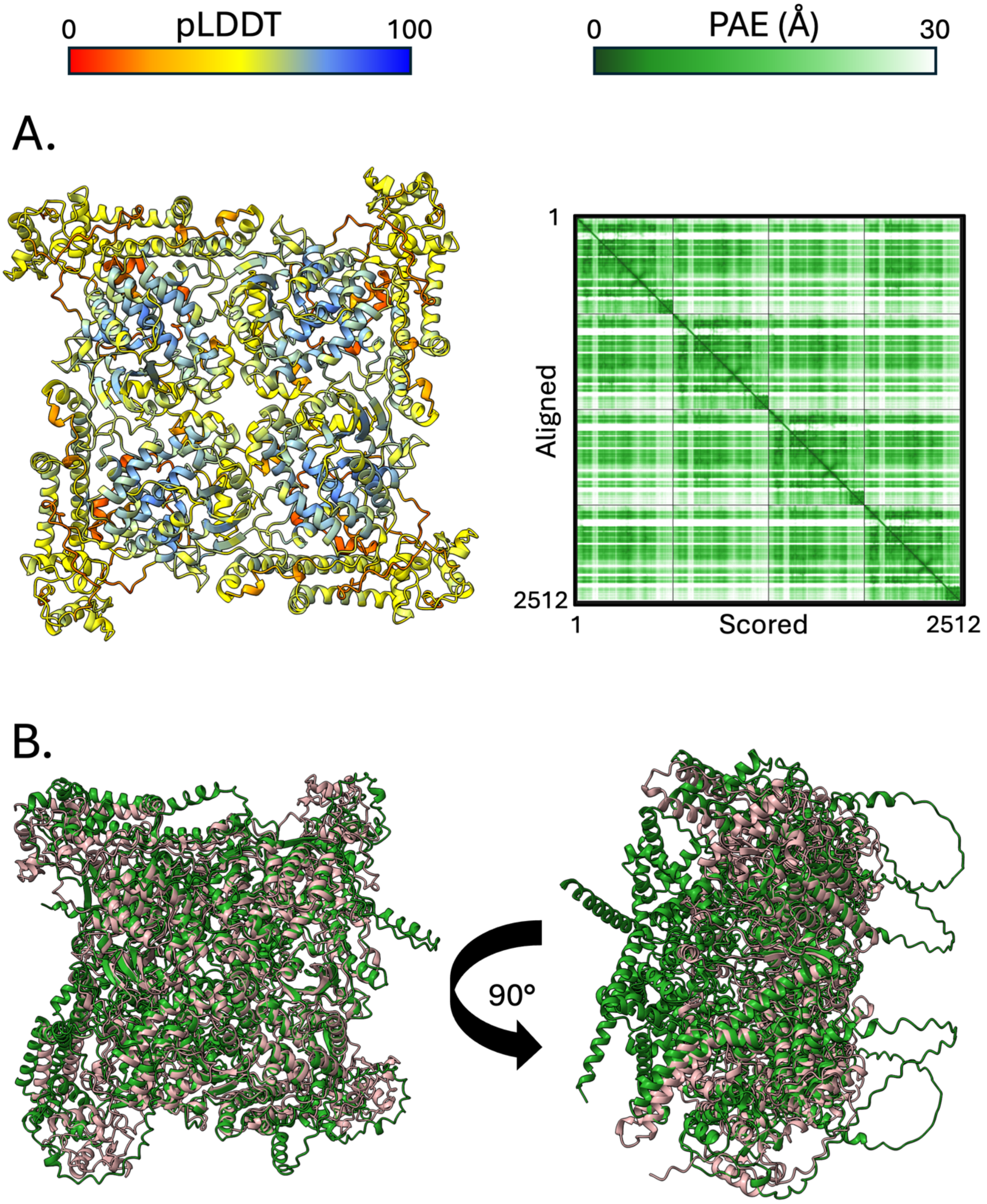

Supplementary Figure 7. Comparison of global folds between tetrameric *Sanctuary* YR prediction and the P1 Cre recombinase (PDB: 3C29). A) Structure of the tetrameric P1 Cre recombinase protein. B) Tetrameric AlphaFold3 prediction of the *Sanctuary* YR protein, see Figure 4 for prediction confidence. C) Superposition of the *Sanctuary* YR tetrameric prediction on top of the Cre tetramer demonstrating similarity between global folds.

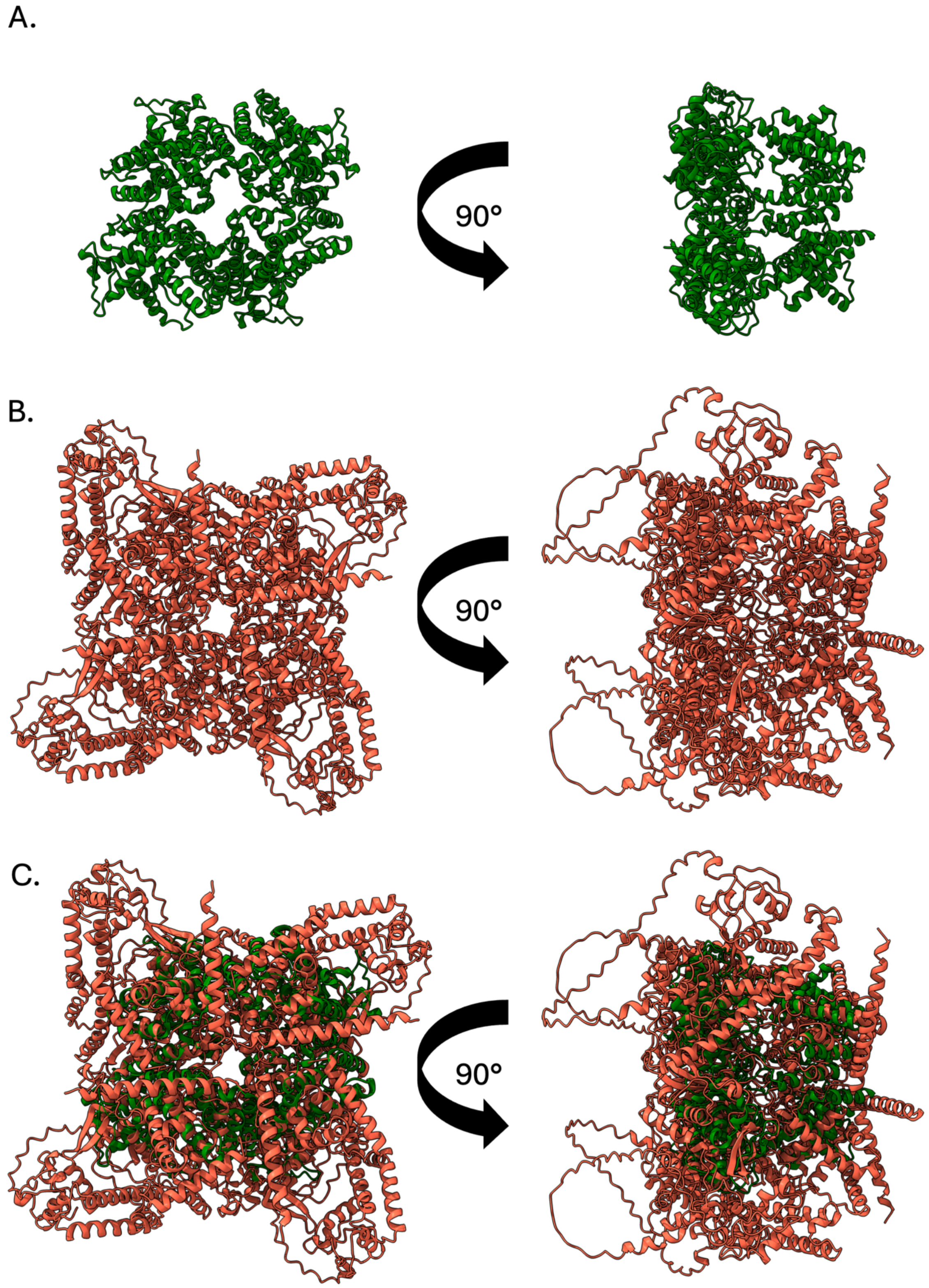

Supplement: Supplemental Figures — Figures S1 to S7. [file mbio.01371-25-s0001.pdf]
